# Distribution and seasonal fluctuations of *Ae*. *aegypti and Ae*. *albopictus* larval and pupae in residential areas in an urban landscape

**DOI:** 10.1371/journal.pntd.0008209

**Published:** 2020-04-20

**Authors:** Jayanthi Rajarethinam, Janet Ong, Zhi-Wei Neo, Lee-Ching Ng, Joel Aik

**Affiliations:** 1 Environmental Health Institute, National Environment Agency, Singapore, Singapore; 2 Environmental Public Health Operations, National Environment Agency, Singapore, Singapore; 3 School of Biological Sciences, Nanyang Technological University, Singapore; 4 School of Public Health and Community Medicine, Faculty of Medicine, University of New South Wales, Level 3, Samuels Building, Botany Road, Keningston, New South Wales, Australia; Universita degli Studi di Pavia, ITALY

## Abstract

Dengue, a vector-borne disease spread by *Aedes* mosquitoes, is a global threat. In the absence of an efficacious dengue vaccine, vector control is the key intervention tool in Singapore. A good understanding of vector habitats is essential to formulate operational strategies. We examined the distribution, long-term trend and seasonality of *Aedes* data collected during regulatory inspections in residences and public areas from 2008 to 2017. We also studied the seasonality of climate factors to understand their influence on the detection of *Aedes*-positive containers. The most frequently reported *Aedes*-positive containers were domestic containers, drains, discarded receptacles, ornamental containers, flower pot plates/trays, plants, gully traps, canvas/plastic sheet, bins, ground puddle, inspection chambers and roof tops/gutters. We found more *Ae*. *aegypti* and *Ae*. *albopictus*-positive containers per inspection in residences and public areas, respectively. The seasonality of *Ae*. *aegypti*-positive containers in residences and public areas coincided with that of mean temperature. However, the seasonality of *Ae*. *albopictus*-positive containers lagged by one month compared to that of mean temperature. Our study demonstrates the seasonal fluctuations of *Aedes*-positive containers in an urban environment. Understanding the distribution and seasonality of *Aedes* breeding helps to facilitate resource planning and community awareness to moderate dengue transmission.

## Author summary

Dengue, a vector-borne disease spread by *Aedes* mosquitoes, is a global threat. In countries where dengue vaccine is not authorized due to safety concerns and is not cost effective, vector control is the key intervention tool. A good understanding of *Aedes* habitats is essential to facilitate resource planning and community awareness to moderate dengue transmission. In this study, we used larvae surveillance data from Singapore, to examine the distribution, long term trend and seasonality of *Aedes* habitats in residences and public areas. We also studied the seasonality of climate factors to understand their influence on the *Aedes* habitats. The most frequently reported *Aedes*-positive containers were domestic containers, drains, discarded receptacles, ornamental containers, flower pot plates/trays, plants, gully traps, canvas/plastic sheet, bins, ground puddle, inspection chambers and roof tops/gutters. Residences had more *Ae*. *aegypti*-positive containers per inspection whereas public areas had more *Ae*. *albopictus*-positive containers per inspection. We also found that mean temperature influenced the detection of *Aedes* habitats. This study demonstrates the seasonal fluctuations of *Aedes*-positive containers in an urban environment.

## Introduction

Dengue fever is transmitted to humans by *Aedes* mosquitoes, mainly *Ae*. *aegypti*, although *Ae*. *albopictus* has also been implicated in several countries [1–5]. It is the fastest growing vector-borne disease in the world with a recent study estimating as many as 390 million dengue infections in a year [6]. The health burden of dengue is disproportionate, with countries in the Asia-Pacific region bearing about three-quarters of the global dengue disease burden [7]. The World Health Organization (WHO) recognizes dengue as a major public health problem and works with relevant countries and development partners to provide strategic guidance for urgent strengthening of vector control as a fundamental approach to reduce disease mobility and mortality rate [8]. With climate change studies indicating the likelihood of an expansion in the geographical areas favouring for dengue transmission, a larger proportion of the global human population is expected to be at risk [9]. An effective vaccine would be an ideal intervention to address the enlarged risk of dengue infections. Although the Dengvaxia dengue vaccine is presently licensed in several settings and several dengue vaccines are in various stages of clinical trials [10–12], there is presently no effective vaccine that can effectively and safely lower the dengue burden of countries with different intensities of dengue incidences. [13]. Vector control, therefore, remains a key strategy for dengue control.

Several studies have examined the distribution and abundance of *Aedes* pupae and larvae (immatures) in household containers in different landscapes for the purpose of planning and managing the vector. For example, recent studies in Kenya, Columbia and Selangor have shown that there are substantial numbers of *Ae*. *aegypti* habitats found in both rural and urban areas [14–16].

Singapore has a dengue control programme that primarily focuses on vector source reduction through surveillance, enforcement, community engagement, careful urban planning and operational research [17]. In this present study, we examined the distribution, long-term trend and seasonality of *Ae*. *aegypti* and *Ae*. *albopictus*-positive containers detected in residences and public areas in Singapore. We also studied the seasonality of climate factors to understand their influence on the detection of *Aedes*-positive containers. Understanding the distribution of *Ae*.*aegypti* and *Ae*. *albopictus*-positive containers detected in regulatory inspections can guide the prioritization of limited vector control resources aimed at reducing arboviral transmission.

## Materials and methods

### Ethics

This study was granted approval by the Environmental Health Institute of the National Environment Agency, Singapore. The study did not involve human participants.

### Study setting

We conducted our study in Singapore (1° 17’N 103° 50’E), a city state with a land area of 724.2 square kilometer, and a population density of 7,804 people per square kilometer, one of the highest population densities in the world [18]. Singapore experiences a tropical climate with abundant rainfall, high and uniform temperatures and high humidity all year round [19]. The study was carried out in residential estates, which comprises both residences and public areas. Residential estates make up 31% of the total land area of Singapore.

Residences are defined as premises under the care of home owners. There are three types of residences: high rise public apartment blocks, private apartment blocks and landed houses. Ninety-five percent of Singapore residents live in apartment blocks, while 5% of them live in landed houses [20]. The public and private residential apartment blocks range from three to 50 storeys, with the majority between 10 and 30 storeys. Landed houses are built by the private sector, and the built up area of each house is on average 2.5 times that of an apartment [21]. Residences are primarily indoor areas, however, a small portion of the total area includes sheltered corridors along the homes of apartment blocks, as well as non-sheltered areas such as gardens of landed houses. Potted plants are common along the sheltered corridors outside homes in apartment blocks. Landed houses typically include external paved and turf areas as well as gardens where plants and ornamental containers are commonly found.

Public areas are defined as open spaces located in residential estates and are accessible to the public. They do not include areas that belong to home owners. The majority of public areas is not sheltered and is directly exposed to the natural elements. However, a small portion of public areas includes sheltered pathways connecting apartment blocks, and sheltered common congregation areas in the residential estate.

### Vector control

As part of Singapore’s dengue control programme, approximately 600 trained public health inspectors from the National Environment Agency (NEA) conduct routine regulatory inspections of indoor premises and outdoor areas to identify and destroy mosquito immatures. All premises are scheduled to be inspected at least once every year, throughout the study period. The inspections of premises are planned at the start of the year and prioritized based on the dengue risk level of their locations. The risk level is determined by several factors such as dengue exposure, population density, and other environmental factors. The present study focuses on *Ae*. *aegypti* and *Ae*. *albopictus* mosquito-positive containers collected in residential estates, hence, mosquito-positive containers detected in other areas such as commercial buildings, construction sites, industrial buildings and other premises (such as schools, places of worship) were excluded from the analyses.

Public health inspectors are trained on inspecting wet containers and collecting mosquito immatures and recording data into the central information database. After receiving permission, surveys are carried out in residences. Majority of the residents grant permission to access their homes. However, in the event that nobody is present, pamphlets that educate residents on how to keep their homes free of mosquitoes, are inserted into homes. Based on the accessibility rate, on average, about half of the residences are surveyed. Majority of the premises are inaccessible because occupants are not present during the time of visit, and not due to refusal of entry. Permission to survey public areas is not required, as NEA authorizes public health inspectors to search and destroy mosquito breeding containers in public areas. At each site, the public health inspector will carefully inspect the area looking for water containers with mosquito immatures. A container is considered positive if a mosquito immature is present. When a container with at least one mosquito immature is found, a three milliliter pipette or specially designed ladle is used to collect water and immatures from the container. The water together with the immatures is transferred to transparent tubes, which can hold up to 12 milliliter of liquid, and labeled with a sample ID. All data pertaining to the collection, such as, the sample identification number, survey date, the type of container, the number of immatures present in the container, and the address where the container is found will be recorded into the central database connected via tablets. Any excess water that has not been collected will be emptied and if emptying the container was not feasible due to the large size, the water will be treated with *Bacillus thuringiensis var*. *israelensis* (BTI) or oiled to destroy the immatures that are not physically removed. At the end of each working day, the tubes with the samples are transported to the Environmental Health Institute (EHI) laboratory for identification of species according to taxonomic keys [22].

### Climate data

Monthly mean temperature, absolute humidity and total rainfall from 2008 to 2017 recorded in Changi weather station in Singapore is obtained from Meteorological Services Singapore.

### Statistical analyses

The monthly number of *Ae*. *aegypti* and *Ae*. *albopictus*-positive containers detected in residences and public areas from 2008 to 2017 was extracted from the database for the analyses. The containers were classified into ten categories based on the frequency of occurrences and the descriptions of the containers are in S1 Text. Containers that contained mosquito immatures that are not *Ae*. *aegypti* and *Ae*. *albopictus* (such as *Culex*. *quinquefasciatus* immatures) were excluded from the analyses for this study. We also retrieved the monthly number of inspections conducted in residences and public areas for the study period.

The monthly ratio of *Ae*. *aegypti* and *Ae*. *albopictus*-positive containers in residences and public areas was calculated by dividing the monthly number of *Ae*. *aegypti* and *Ae*. *albopictus*-positive containers in residences and public areas by the total number of inspections carried out in residences and public areas, respectively. A monthly ratio was chosen so as average out random noise. All premises within a premises types are similar to each other in terms of land area. Hence, the assumption we take is that the time taken for each inspection within a premises type is comparable.

Descriptive analyses were used to explore the data. We tabled the monthly number of *Ae*. *aegypti* and *Ae*. *albopictus*-positive containers detected in residences and public areas for the ten categories of containers. The monthly ratio of *Ae*. *aegypti* and *Ae*. *albopictus*-positive containers in residences and public areas was plotted over time. As the monthly mean ratio of *Ae*. *aegypti*-positive containers and *Ae*. *albopictus*-positive containers in residences as well as public areas were normally distributed, a paired t-test was used to compare the differences. We derived the trend and seasonality components of the monthly ratio of *Ae*. *aegypti* and *Ae*. *albopictus*-positive containers in separate time series models for each species, using the “ts” and “decompose” package implemented in the R statistical language [23] for residences and public areas. We also assessed the seasonality of the monthly climate factors in separate time series models in order to see if the seasonality of climate factors coincided with that of the *Aedes*-positive containers. Using Pearson cross correlation tests, we compared the seasonality of climate factors with that of *Aedes*-positive containers to determine the lag at which the correlation is the strongest. Generalized additive regression models were built for each premises types, that considers species and time components as the variables, using the “gam” package. Splines are added, where relevant, for smoothing. The models compare the trend of ratios between each species for each premises types. Statistical analyses were performed using R software version 3.6.1 [23]. Statistical significance was assessed at the 5% level.

## Results

### Taxonomic identification

There were 151,512 inspections conducted in residences (58.8%) and public areas (41.2%) from 2008 to 2017. Containers with mixed (*Ae*. *aegypti* and *Ae*. *albopictus*) breeding contributed to 3% of the total number of *Aedes*-positive containers. These data will be excluded from the study. A total of 149,184 *Aedes*-positive containers were found. Of these, 64,375 (43.2%) were *Ae*. *aegypti*-positive containers and 84,809 (56.8%) were *Ae*. *albopictus*-positive containers. Among the *Ae*. *aegypti*-positive containers detected, 83.3% were found in residences, while 51.4% of all *Ae*. *albopictus*-positive containers were detected in public areas.

### *Ae*. *aegypti* and *Ae*. *albopictus*-positive containers

**Fig 1:**
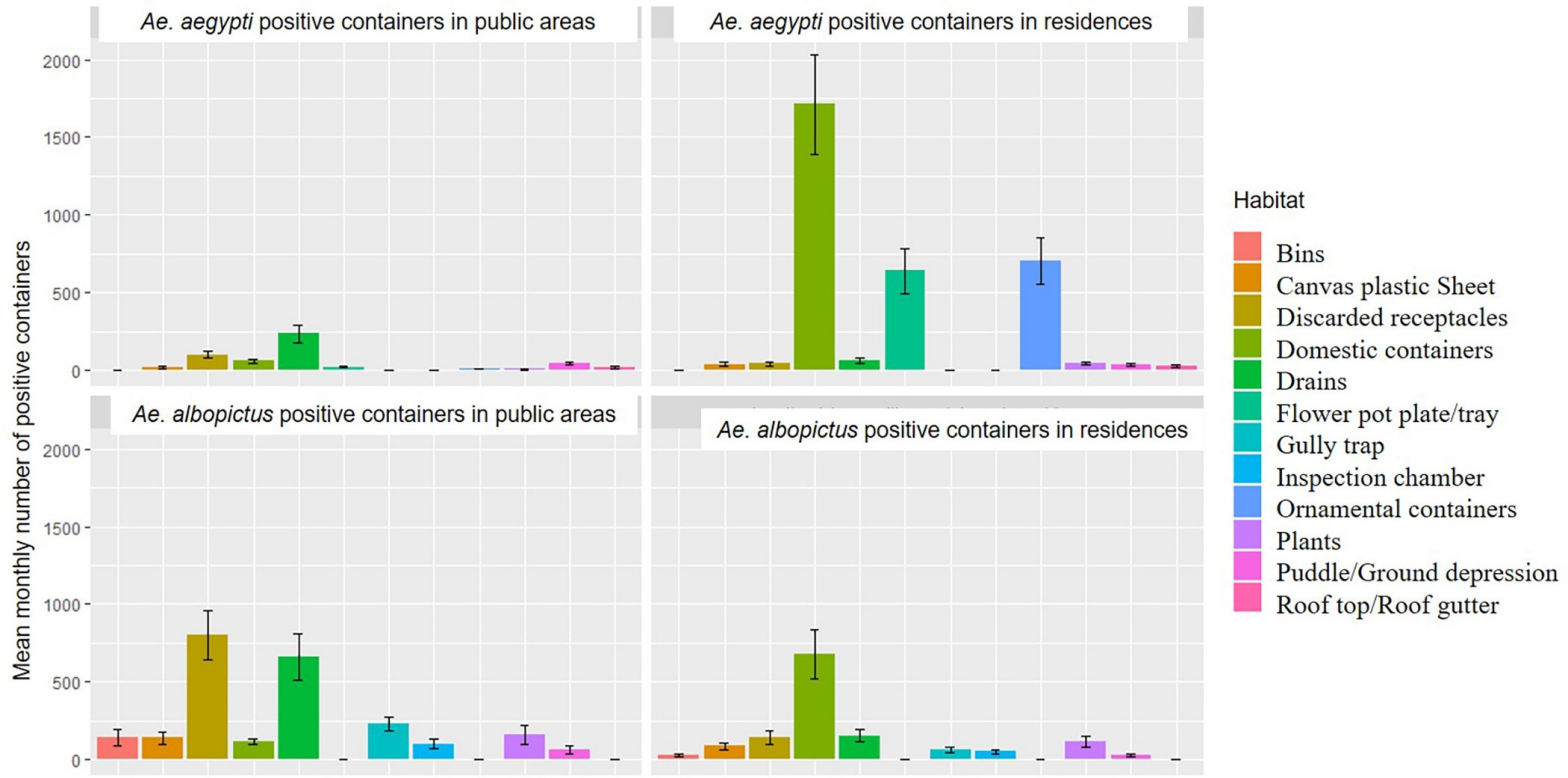
Mean monthly number of *Ae*. *aegypti* and *Ae*. *albopictus-*positive containers detected in residences and public areas.

The most frequently reported categories of *Aedes*-positive containers were domestic containers, ornamental containers, flower pot plates/trays, drains, plants, discarded receptacles, canvas sheet/plastic sheet, puddle/ground depression, roof top/roof gutters, discarded receptacles, gully traps, inspection cover chambers, bins and “others” (S1 Text). Among the top ten categories of *Ae*. *aegypti* and *Ae*. *albopictus*-positive containers, there were six containers that were common to both species. The nine most frequently reported categories of *Ae*. *aegypti*-positive containers accounted for 74.4% and 56.5% of total *Ae*. *aegypti*-positive containers found in residences and public areas, respectively. In the case of *Ae*. *albopictus*-positive containers, the top nine categories of *Ae*. *albopictus-*positive containers accounted for 57.8% and 72.3% of all *Ae*. *albopictus*–positive containers in residences and public areas, respectively. The mean weekly number of *Ae*. *aegypti* and *Ae*. *albopictus*-positive containers in residences and public areas are shown in Fig 1.

**Fig 2:**
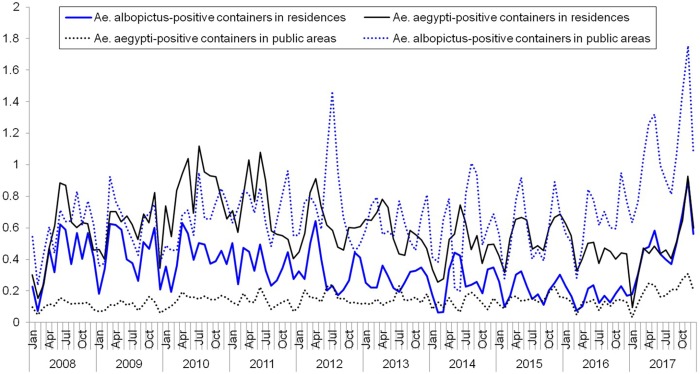
Monthly ratio of *Ae*. *aegypti and Ae*. *albopictus*-positive containers in residences and public areas from 2008 to 2017.

Over the ten-year study period, *Ae*. *aegypti* and *Ae*. *albopictus*-positive containers in residences and public areas exhibited different temporal trends (Fig 2). The mean ratio of *Ae*. *aegypti*-positive containers was significantly higher than the mean ratio of *Ae*. *albopictus*-positive containers in residences (difference in mean = 0.25, 95% CI = 0.21 to 0.39, p<0.05). In contrast, the mean ratio of *Ae*. *albopictus*-positive containers was significantly higher than the mean ratio of *Ae*. *aegypti*-positive containers in public areas (difference in mean = 0.55, 95% CI = 0.50 to 0.60, p<0.05).

### Time-series analyses of monthly *Aedes*-positive containers per inspection in residences and public areas

**Fig 3:**
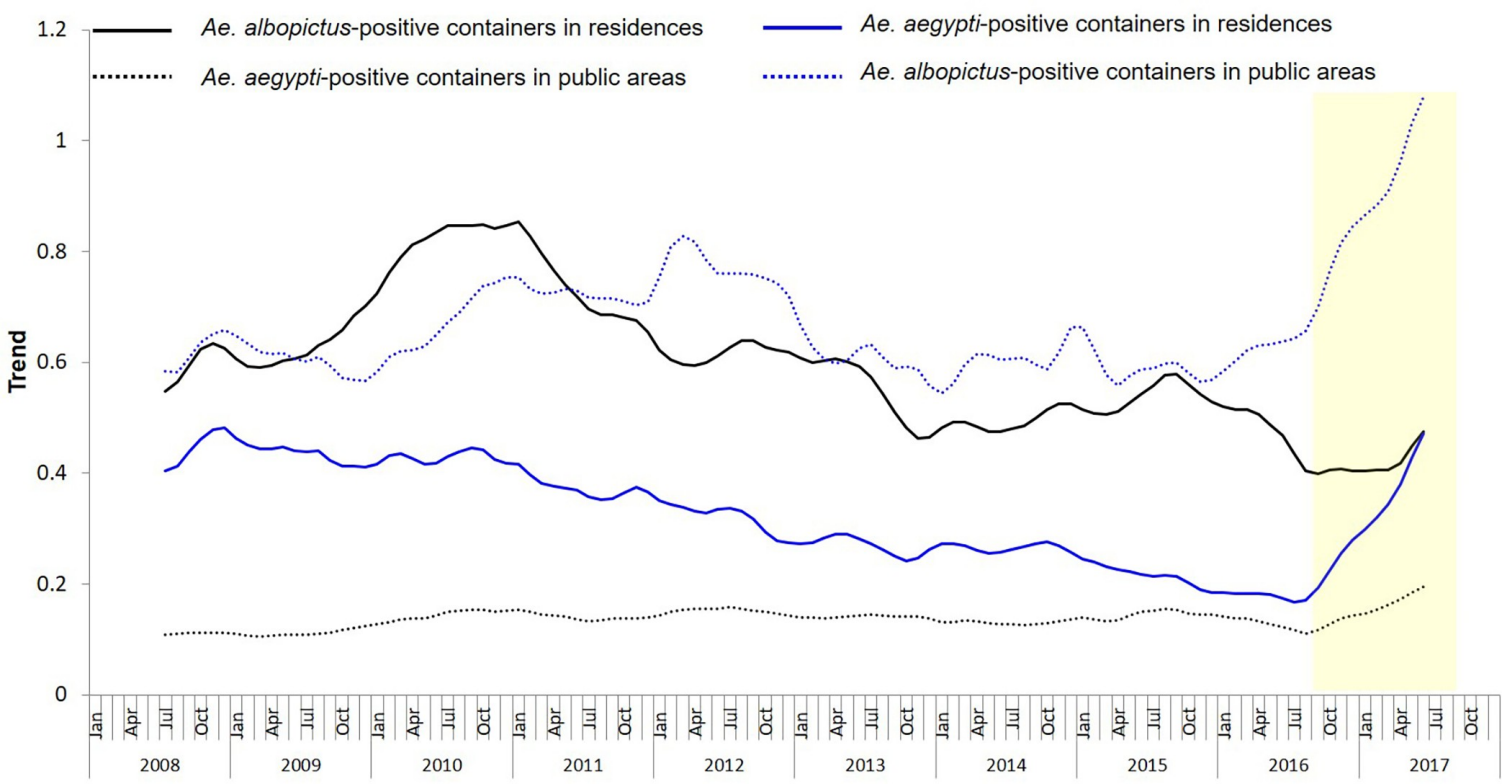
The trend of ratio of *Ae*. *aegypti and Ae*. *albopictus*-positive containers in residences and public areas from 2008 to 2017. An upward trend for all lines were observed from Sep 2016, as highlighted in the yellow shaded region.

The monthly trend of the ratio of *Ae*. *aegypti* and *Ae*. *albopictus*-positive containers for both residences and public areas was plotted over time (Fig 3). There was an increasing trend in the ratio of *Ae*. *aegypti*-positive containers in residences from 2008 to 2010, after which there was a decreasing trend till September 2016. An upward trend was observed in the ratio of *Ae*. *albopictus*-positive containers in public areas from 2010 to mid 2012, after which the trend was stable until September 2016, There was a decreasing trend in the ratio of *Ae*. *albopictus*-positive containers in residences from 2008 to September 2016. The trend of ratio of *Ae*. *albopictus*-positive containers in public areas remained relatively stable, till September 2016. There was an upward trend in the monthly ratio of *Ae*. *aeygpti and Ae*. *albopictus*-positive containers in both residences and public areas from September 2016.

The rate of increase in the trend of *Ae*. *albopictus*-positive breeding was greater than that of *Ae*. *aegypti*-positive breeding in residences as well as public areas (Table 1).

**Table 1:** Monthly rate of increase of trend of the rate of *Aedes*-positive containers in residences and public areas from September 2016 to December 2017. The results are from the additive regression models created to compare the trends between *Ae*. *aegypti*-positive containers and *Ae*. *albopictus*-positive containers in residences and public areas.

Trend line	Mean	95% CI	p-value
Rate of increase of *Ae*. *aegypti*-positive containers per inspection in residences	0.006	0.005–0.012	<0.05
Rate of increase of *Ae*. *albopictus*-positive containers per inspection in residences	0.030	0.026–0.110	<0.05
Rate of increase of *Ae*. *aegypti*-positive containers per inspection in public areas	0.008	0.002–0.014	<0.05
Rate of increase of *Ae*. *albopictus*-positive containers per inspection in public areas	0.038	0.033–0.120	<0.05

**Fig 4:**
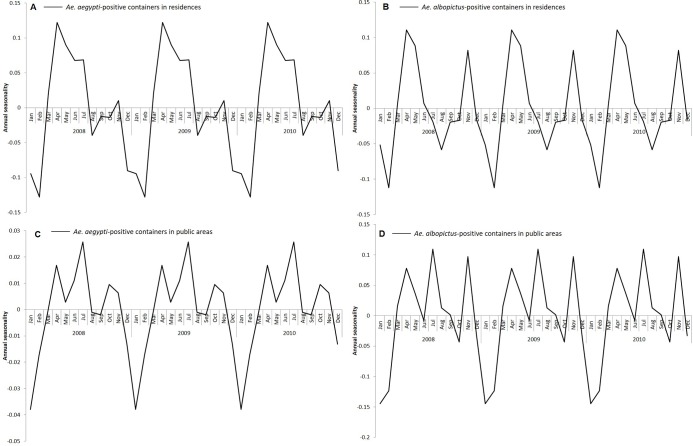
The seasonality of *Aedes*-positive containers in residences and public areas from 2008 to 2017. The first 3 years are shown for easier visualization. The seasonality of ratio of (A) *Ae*. *aegypti*-positive containers in residences (B) *Ae*. *albopictus*-positive containers in residences (C). *aegypti*-positive containers in public areas (D) *Ae*. *albopictus*-positive containers in public areas.

There were seasonal fluctuations in the ratio of *Aedes*-positive containers in residences and public areas (Fig 4). For residences, we observed two peaks in the ratio of *Ae*. *aegypti* and *Ae*. *albopictus*-positive containers in April and November, although the magnitude of the peak in November was smaller for *Ae*. *aegypti* compared to *Ae*. *albopictus*. For public areas, however, an additional peak occurred in July in the ratio of both *Ae*. *aegypti* and *Ae*. *albopictus*-positive containers. The seasonal peak in the ratio of *Ae*. *aegypti* and *Ae*. *albopictus*-positive containers in residences and public areas was lowest in February. The seasonal and trend components of *Aedes*-positive containers are found in S2 Text.

### Time-series analyses of monthly mean temperature, total rainfall and absolute humidity

**Fig 5:**
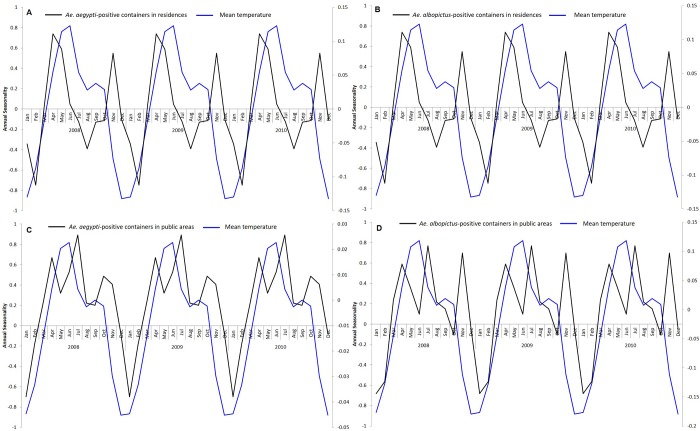
The seasonality of mean temperature and *Ae*. *aegypti* and *Ae*. *albopictus*-positive containers from 2008 to 2017. The first 3 years are shown for easier visualization. The seasonality of mean temperature plotted with the seasonality of (A) *Ae*. *aegypti*-positive containers in residences (B) *Ae*. *albopictus*-positive containers in residences (C) *Ae*. *aegypti*-positive containers in public areas (D) *Ae*. *albopictus*-positive containers in public areas.

We observed seasonal fluctuations in weather parameters over the study period. The mean temperature peaks from April to June, and again a slight peak was observed in September. The mean temperature in December to February was generally lower. The highest correlation between the seasonality of *Ae*. *aegypti-*positive containers and mean temperature in residences (r = 0.81) and public areas (r = 0.73) had no time lag, while, in the case of *Ae*. *albopictus*-positive containers, the highest correlation in residences (r = 0.41) and public areas (r = 0.65), was observed when *Ae*. *albopictus*-positive containers lagged mean temperature by 1 month (p<0.05). (Fig 5)

**Fig 6:**
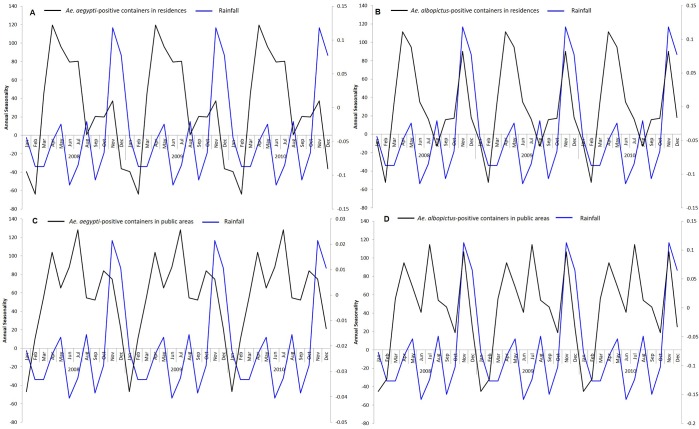
The seasonality of rainfall and *Ae*. *aegypti* and *Ae*. *albopictus*-positive containers from 2008 to 2017. The first 3 years are shown for easier visualization. The seasonality of rainfall plotted with the seasonality of (A) *Ae*. *aegypti*-positive containers in residences (B) *Ae*. *albopictus*-positive containers in residences (C) *Ae*. *aegypti*-positive containers in public areas (D). *albopictus*-positive containers in public areas.

In contrast, the seasonal peak in rainfall occurred in November and was at its lowest in June (Fig 6). There was only weak correlation between the seasonality of rainfall and that of *Aedes*-positive containers in residences and public areas. However, the timing of the seasonal peak in rainfall coincided with the year-end peaks in *Ae*. *albopictus*-positive containers in residences and public areas.

**Fig 7:**
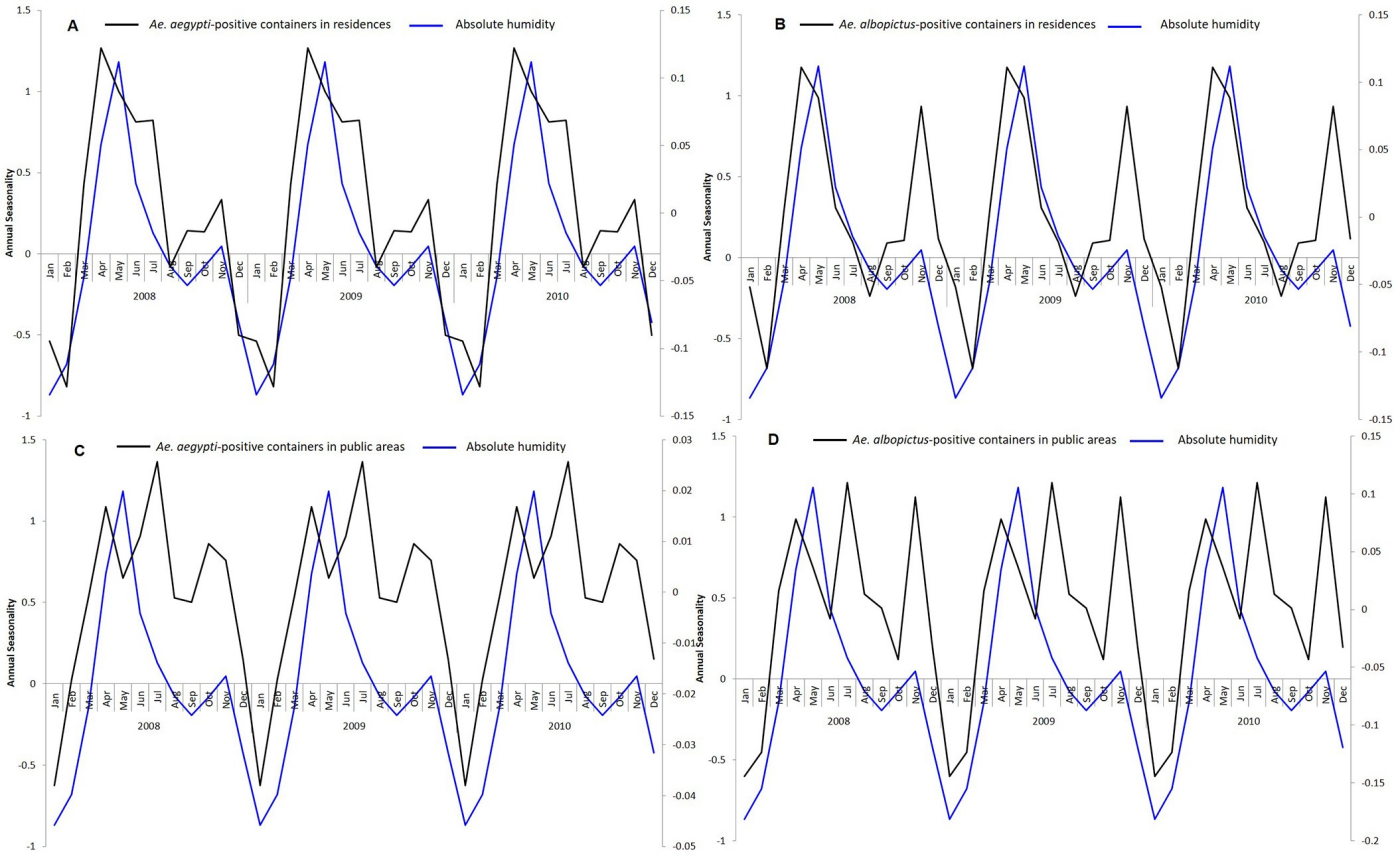
The seasonality of absolute humidity and *Ae*. *aegypti* and *Ae*. *albopictus*-positive containers from 2008 to 2017. The seasonality of absolute humidity plotted with the seasonality of (A) *Ae*. *aegypti*-positive containers in residences (B) *Ae*. *albopictus*-positive containers in residences (C) *Ae*. *aegypti*-positive containers in public areas (D) *Ae*. *albopictus*-positive containers in public areas.

The timing of the peaks and troughs of absolute humidity was similar to that of *Aedes*-positive containers (Fig 7). The seasonal fluctuations of *Ae*. *aegypti*-positive containers in residences (r = 0.89) and public areas (r = 0.70) coincided with seasonal fluctuations of absolute humidity.

## Discussion

We examined the temporal distribution of *Ae*. *aegypti* and *Ae*. *albopictus-*positive containers detected during regulatory inspections in residences and public areas for the past decade in Singapore. We found more *Ae*. *aegypti-*positive containers in residences and more *Ae*. *albopictus*-positive containers in public areas. This finding is consistent with the last study on *Aedes* larval habitats in Singapore, conducted in 1960s, which showed that the majority of *Ae*. *aegypti* and *Ae*. *albopictus* larval habitats were found in homes and outdoor settings, respectively [24]. Similar results were also reported in other countries such as Sarawak [25], Peninsular Malaysia [26] and South Sulawesi, Indonesia [27]. This finding is consistent with existing knowledge that *Ae*. *aegypti* are highly anthropophilic mosquitoes that are usually found in indoor areas with close proximity to humans whereas *Ae*. *albopictus* mosquitoes prefers natural habitats [28,29]. *Ae*. *aegypti* mosquitoes are competent dengue vectors [1–4]. Therefore, in the presence of *Ae*. *aegypti* population and high human density, there could be an increased risk of dengue transmission inside human dwellings. A previous study in Singapore has also shown that areas with higher *Ae*. *aegypti* proportion are likely to be more vulnerable to dengue outbreaks [30]. Hence, to mitigate dengue transmission, it is necessary for residents to continue to carry out mosquito control activities in homes.

A consistent upward trend in the ratio of *Ae*. *aegypti* and *Ae*. *albopictus*-positive containers in both residences and public areas was observed from September 2016. It is possible that mosquito control practices adopted by the general public were not sufficient over the years, thus, resulting in an increase in the mosquito population. This observation reinforces the need to further enhance public communications to urge individuals to take ownership in carrying out mosquito-control activities. Nevertheless, it is reassuring that the increase in the ratio of *Ae*. *aegypti* was less pronounced than *Ae*. *albopictus*.

There were seasonal fluctuations in the rate of *Aedes-*positive containers in residences and public areas. However, within residences and public areas, the seasonality of the rate of *Ae*. *aegypti* and *Ae*. *albopictus-*positive containers was similar. This suggests that *Ae*. *aegypti* and *Ae*. *albopictus*-positive containers within a particular premises type are influenced by common external factors. The seasonal fluctuations of *Ae*. *aegypti*-positive containers in residences and public areas coincided with seasonal fluctuations of mean temperature. However, there was a one-month lag in *Ae*. *albopictus*-positive containers when comparing the seasonal fluctuations of *Ae*. *albopictus*-positive containers in residences and public areas with the season fluctuations of mean temperature. It is likely that temperature influences the growth of *Aedes* larvae in both premises types. A similar study conducted in Cairns, Australia reported that the *Ae*. *aegypti* population lags maximum temperature between short (0–6 weeks) and long (0–30) weeks. [31]. A more recent study showed that the development time from hatching to adult emergence was shorter at higher temperatures [32], which could explain the increase in the number of mosquito-positive containers detected when temperature rises. Temperature trends can be used as a guide for shaping our vector control programme. Intervention efforts can be enhanced when the temperature rises, so as to control the proliferation of *Aedes* mosquito population. Interestingly, we noticed that the year-end peak in *Ae albopictus*-positive containers was higher than the initial peak for both residences and public areas, compared to *Ae*. *aegypti*-positive containers. The timing of the year end peak coincided with the seasonal peak in the total rainfall suggesting that rainfall has a larger effect on *Ae*. *albopictus* compared to *Ae*. *aegypti*. This could be due to *Ae*. *albopictus* preferring natural habitats, which are more prone to rainfall accumulation, compared to *Ae*. *aegypti* [33,34].

### Study strengths and limitations

We analysed a nationally representative data set collected over a decade. Understanding the long term trend and seasonality of *Aedes*-positive containers allows us to make informed decisions in enhancing vector control policies to achieve maximum impact. There are a few study limitations. Firstly, public health inspectors are trained to look out for containers that are known to breed *Aedes* mosquitoes. This could have skewed the list of most frequently reported *Aedes*-positive containers. Secondly, there might be cryptic breeding sites that are difficult to identify or locate by public health inspectors, such as elevated sites or subterranean sites. Such sites might have been missed out in the list of most frequently reported habitats, and therefore, the number of *Aedes*-positive containers may be underestimated due to the lack of knowledge of these cryptic sites. Another limitation is that the time taken to inspect a residence is not similar to that of a public area, as the land area of a public area is larger and there is higher probability of detecting more potential breeding habitats. The last limitation is that there could be a possible impact of larviciding and source removal which can affect the presence of positive containers in the subsequent inspection, and we are unable to account for this in the computation of the rates. However, we expect little effect of this limitation on the study findings, as a previous study on the regulatory inspections in Singapore has shown that proportion of residences with repeated *Aedes*-positive containers detected were low [35].

Our study showed that there are more *Ae*. *aegypti-*positive containers in residences and more *Ae*. *albopictus*-positive containers in public areas. The seasonality of *Ae*. *aegypti*-positive containers in residences and public areas coincided with that of mean temperature. However, the seasonality of *Ae*. *albopictus*-positive containers lagged by one month compared to that of mean temperature. Understanding the distribution and seasonality of *Aedes* breeding helps to facilitate resource planning and community awareness to moderate dengue transmission.

## Supporting information

S1 Text Categorization and definitions of the most frequently reported *Aedes*-breeding containers. (DOCX)

S2 Text The seasonal and trend components of *Aedes*-positive containers in residences and public areas. (XLSX)

## References

[1] Gratz NG. Critical review of the vector status of Aedes albopictus. Medical and veterinary entomology. 2004 9;18(3):215–27. 10.1111/j.0269-283X.2004.00513.x 15347388

[2] Gubler DJ. Dengue and dengue hemorrhagic fever. Clinical microbiology reviews. 1998 7 1;11(3):480–96. 9665979 10.1128/cmr.11.3.480 PMC88892

[3] Gubler DJ. Resurgent vector-borne diseases as a global health problem. Emerging infectious diseases. 1998 7;4(3):442 10.3201/eid0403.980326 9716967 PMC2640300

[4] Lambrechts L, Scott TW, Gubler DJ. Consequences of the expanding global distribution of Aedes albopictus for dengue virus transmission. PLoS neglected tropical diseases. 2010 5 25;4(5):e646 10.1371/journal.pntd.0000646 20520794 PMC2876112

[5] Luo L, Jiang LY, Xiao XC, Di B, Jing QL, Wang SY, Tang JL, Wang M, Tang XP, Yang ZC. The dengue preface to endemic in mainland China: the historical largest outbreak by Aedes albopictus in Guangzhou, 2014. Infectious diseases of poverty. 2017 12;6(1):148 10.1186/s40249-017-0352-9 28934991 PMC5609019

[6] Velayudhan R (2010). Dengue: the fastest growing mosquito-borne disease in the world. World Health Organisation https://www.who.int/denguecontrol/news/integrated_media_2010_Dengue_vs_malaria/en/ Accessed at: 19 Dec 2018

[7] Bhatt S, Gething PW, Brady OJ, Messina JP, Farlow AW, Moyes CL, Drake JM, Brownstein JS, Hoen AG, Sankoh O, Myers MF. The global distribution and burden of dengue. Nature. 2013 4;496(7446):504 10.1038/nature12060 23563266 PMC3651993

[8] Geneva: World Health Organization (2018). Global vector control response 2017–2030. Licence: CC BY-NC-SA 3.0 IGO https://www.who.int/vector-control/publications/global-control-response/en/ Accessed at: 19 Dec 2018

[9] Hales S, De Wet N, Maindonald J, Woodward A. Potential effect of population and climate changes on global distribution of dengue fever: an empirical model. The Lancet. 2002 9 14;360(9336):830–4. 10.1016/S0140-6736(02)09964-6 12243917

[10] Schwartz LM, Halloran ME, Durbin AP, Longini IM Jr. The dengue vaccine pipeline: Implications for the future of dengue control. Vaccine. 2015 6 26;33(29):3293–8. 10.1016/j.vaccine.2015.05.010 25989449 PMC4470297

[11] Pang EL, Loh HS. Towards development of a universal dengue vaccine–How close are we?. Asian Pacific journal of tropical medicine. 2017 3 1;10(3):220–8. 10.1016/j.apjtm.2017.03.003 28442105

[12] World Health Organization Research and Development: Immunization, Vaccines and Biologicals, 5 Dec 2017. 2017. https://www.who.int/immunization/research/en/ Accessed at 30 Jan 2019

[13] Wilder-Smith A, Hombach J, Ferguson N, Selgelid M, O'Brien K, Vannice K, Barrett A, Ferdinand E, Flasche S, Guzman M, Novaes HM. Deliberations of the strategic advisory group of experts on immunization on the use of CYD-TDV dengue vaccine. The Lancet infectious diseases. 2019 1 1;19(1):e31–8. 10.1016/S1473-3099(18)30494-8 30195995

[14] Overgaard HJ, Olano VA, Jaramillo JF, Matiz MI, Sarmiento D, Stenström TA, Alexander N. A cross-sectional survey of Aedes aegypti immature abundance in urban and rural household containers in central Colombia. Parasites & vectors. 2017 12;10(1):356 10.1186/s13071-017-2295-1 28750651 PMC5530958

[15] Ngugi HN, Mutuku FM, Ndenga BA, Musunzaji PS, Mbakaya JO, Aswani P, Irungu LW, Mukoko D, Vulule J, Kitron U, LaBeaud AD. Characterization and productivity profiles of Aedes aegypti (L.) breeding habitats across rural and urban landscapes in western and coastal Kenya. Parasites & vectors. 2017 12;10(1):331 10.1186/s13071-017-2271-9 28701194 PMC5508769

[16] Chen CD, Benjamin S, Saranum MM, Chiang YF, Lee HL, Nazni WA, Sofian-Azirun M. Dengue vector surveillance in urban residential and settlement areas in Selangor, Malaysia. Tropical biomedicine. 2005;22(1):39–43. 16880752

[17] Hapuarachchi HC, Koo C, Rajarethinam J, Chong CS, Lin C, Yap G, Liu L, Lai YL, Ooi PL, Cutter J, Ng LC. Epidemic resurgence of dengue fever in Singapore in 2013–2014: a virological and entomological perspective. BMC infectious diseases. 2016 12;16(1):300 10.1186/s12879-016-1606-z 27316694 PMC4912763

[18] Population and Population Structure. Department of Statistics Singapore. 27 Sep 2018. 2018. https://www.singstat.gov.sg/find-data/search-by-theme/population/population-and-population-structure/latest-data. Accessed at 30 Jan 2019.

[19] Climate of Singapore. Meteorological Service Singapore. 2019. http://www.weather.gov.sg/climate-climate-of-singapore/. Accessed at 30 Jan 2019.

[20] Households. Department of Statistics Singapore. 9 May 2018. 2018 https://www.singstat.gov.sg/find-data/search-by-theme/households/households/latest-data. Accessed at 30 Jan 2019.

[21] Housing around the world. Research and articles made by teoalida. Housing in Singapore. Sep 2018. 2018 https://www.teoalida.com/world/singapore/. Accessed at 30 Jan 2019.

[22] Chan A, Chiang LP, Hapuarachchi HC, Tan CH, Pang SC, Lee R, Lee KS, Ng LC, Lam-Phua SG. DNA barcoding: complementing morphological identification of mosquito species in Singapore. Parasites & vectors. 2014 12;7(1):569 10.1016/0003-9861(89)90518-3 25498759 PMC4282734

[23] Team, R.D.C. R: A language and Environment for Statistical Computing, in R foundation for Statisitcal Computing. 2008: Vienna, Austria.

[24] Chan KL, Ho BC, Chan YC. Aedes aegypti (L.) and Aedes albopictus (Skuse) in Singapore city: 2. Larval habitats. Bulletin of the World Health Organization. 1971;44(5):629 5316746 PMC2427856

[25] Seng CM, Jute N. Breeding of Aedes aegypti (L.) and Aedes albopictus (Skuse) in urban housing of Sibu town, Sarawak. Southeast Asian Journal of Tropical Medicine and Public Health. 1994 9;25:543–. 7777923

[26] Rozilawati H, Tanaselvi K, Nazni WA, Masri SM, Zairi J, Adanan CR, Lee HL. Surveillance of Aedes albopictus Skuse breeding preference in selected dengue outbreak localities, peninsular Malaysia. Trop Biomed. 2015 3 1;32(1):49–64. 25801254

[27] Ishak H, Miyagi I, Toma T, Kamimura K. Breeding habitats of Aedes aegypti (L) and Aedes. albopictus (Skuse) in villages of Barru, South Sulawesi, Indonesia. The Southeast Asian journal of tropical medicine and public health. 1997 12;28(4):844–50. 9656413

[28] Kraemer MU, Sinka ME, Duda KA, Mylne AQ, Shearer FM, Barker CM, Moore CG, Carvalho RG, Coelho GE, Van Bortel W, Hendrickx G. The global distribution of the arbovirus vectors Aedes aegypti and Ae. albopictus. elife. 2015 6 30;4:e08347 10.7554/eLife.08347 26126267 PMC4493616

[29] Higa Y. Dengue vectors and their spatial distribution. Tropical medicine and health. 2011;39(4SUPPLEMENT):S17–27. 10.2149/tmh.2011-S04 22500133 PMC3317606

[30] Ong J, Liu X, Rajarethinam J, Yap G, Ho D, Ng LC. A novel entomological index, Aedes aegypti Breeding Percentage, reveals the geographical spread of the dengue vector in Singapore and serves as a spatial risk indicator for dengue. Parasites & vectors. 2019 12;12(1):17. PMC6325748 30621762 10.1186/s13071-018-3281-y PMC6325748

[31] Duncombe J, Clements A, Davis J, Hu W, Weinstein P, Ritchie S. Spatiotemporal patterns of Aedes aegypti populations in Cairns, Australia: assessing drivers of dengue transmission. Tropical Medicine & International Health. 2013 7;18(7):839–49. 10.1111/tmi.12115 23617766

[32] Reinhold JM, Lazzari CR, Lahondère C. Effects of the environmental temperature on Aedes aegypti and Aedes albopictus mosquitoes: a review. Insects. 2018 12;9(4):158 10.3390/insects9040158 30404142 PMC6316560

[33] Chareonviriyaphap T, Akratanakul P, Nettanomsak S, Huntamai S. Larval habitats and distribution patterns of Aedes aegypti (Linnaeus) and Aedes albopictus (Skuse), in Thailand. Southeast Asian Journal of Tropical Medicine and Public Health. 2003 9;34(3):529–35. 15115122

[34] Dieng H, Rahman GS, Hassan AA, Salmah MC, Satho T, Miake F, Boots M, Sazaly A. The effects of simulated rainfall on immature population dynamics of Aedes albopictus and female oviposition. International journal of biometeorology. 2012 1 1;56(1):113–20. 10.1007/s00484-011-0402-0 21267602

[35] Aik J, Neo ZW, Rajarethinam J, Chio K, Lam WM, Ng LC. The effectiveness of inspections on reported mosquito larval habitats in households: A case-control study. PLoS neglected tropical diseases. 2019 6 26;13(6):e0007492 10.1371/journal.pntd.0007492 31242192 PMC6615626

