# Supplementary material for: Distribution and seasonal fluctuations of Ae. aegypti and Ae. albopictus larval and pupae in residential areas in an urban landscape
Source: PLoS Negl Trop Dis. 2020 Apr 20;14(4):e0008209. doi: 10.1371/journal.pntd.0008209 (PMC7192508; doi:10.1371/journal.pntd.0008209)
Supplement: S1 Text — (DOCX) [file pntd.0008209.s001.docx]

**S1 Appendix**

*Categorization of containers*

*Ae. aegypti*-positive containers and Ae. albopictus-positive containers are both categories into ten categories based on the frequency of occurrences. The top ten categories for *Ae. aegypti*-positive containers are domestic containers, ornamental containers, flower pot plates/trays, drains, plants, discarded receptacles, canvas sheet/plastic sheet, puddle/ground depression and roof top/roof gutters. The top ten categories for *Ae. albopictus*-positive containers are canvas sheet/ plastic sheet, domestic containers, drains, discarded receptacles, plants, gully traps, inspection cover chambers, puddle/ground depression and bins. The tenth category was “others”, which comprised all other *Aedes*-positive containers.

*Definition of containers*

Domestic containers include containers that are commonly used for household purposes such as pails, plastic food containers, bottles, plates, cans etc. Ornamental containers include containers that are commonly used for decorative purposes such as vases and urns. Bins include refuse bins and joss paper/incense bins that are commonly placed in the ground area surrounding high rise buildings. Drains include all types of drains such as covered drains, open drains perimeter drains along the corridors of high rise apartments. Inspection chambers are structures installed underground for sewerage/sanitary and rain water pipe inspection. Gully traps are U-shaped pipes that form a basin in the ground which receives wastewater before it is discharged into the drain or sewer. Some examples of containers categorized under “others” include toys, water fountains, toilet bowl cisterns, water tanks etc. The list of containers is non-exhaustive.
